# Impact of a comprehensive two-year research capacity intervention with sexual and reproductive health researchers in Sub-Saharan Africa

**DOI:** 10.1186/s12978-025-02047-5

**Published:** 2025-05-31

**Authors:** Julie M. Buser, Olive Tengera, Charley Jiang, Edward Kumakech, Rachel Gray, Madeleine Mukeshimana, Gerard Kaberuka, Marie Laetitia Ishimwe Bazakare, Jackline Ayikoru, Diomede Ntasumbumuyange, Tamrat Endale, Pebalo Francis Pebolo, Anna Grace Auma, Ella August, Yolanda R. Smith

**Affiliations:** 1 Center for International Reproductive Health Training (CIRHT), University of Michigan, Ann Arbor, MI 48105 USA; 2 College of Medicine and Health Sciences, University of Rwanda, Kigali, Rwanda; 3 Department of Obstetrics and Gynecology, University of Michigan, Ann Arbor, MI 48105 USA; 4 Department of Nursing and Midwifery, Lira University, Lira, Uganda; 5 Department of Reproductive Health, Gulu University, Gulu, Uganda; 6 Department of Obstetrics and Gynecology, University of Rwanda, Kigali, Rwanda; 7 Department of Obstetrics and Gynecology, College of Medicine and Health Science, School of Medicine and Pharmacy, University Teaching Hospital of Kigali, Kigali, Rwanda; 8 Department of Epidemiology, University of Michigan School of Public Health, Ann Arbor, MI 48105 USA; 9 PREPSS (Pre-Publication Support Service), University of Michigan, Ann Arbor, MI 48105 USA

**Keywords:** Research training intervention, Clinical research appraisal inventory (CRAI) -12, Research competence, Pre-post assessment, Clinical research, Training effectiveness, Sub-Saharan Africa, Women’s health, OBGYN, Nursing and midwifery

## Abstract

**Background:**

Research training in Sub-Saharan Africa is crucial for strengthening the capacity of healthcare professionals and researchers to address specific sexual and reproductive health (SRH) challenges within their communities. Interventions that enhance research capacity and foster a culture of innovation within existing structures offer a practical and economical strategy capable of addressing national and sub-national SRH needs. This study evaluated an intervention to enhance reproductive health research skills to assess research competence changes among participants.

**Methods:**

A pre-post intervention design was employed. Pre- and post-program assessments were conducted using the Clinical Research Appraisal Inventory (CRAI)-12 scale to establish baseline and endline levels of research competence. Descriptive statistics, chi-squared and Kruskal Wallis tests were used to analyze data. Our data interpretation is guided by the Social Cognitive Career Theory.

**Results:**

Faculty members and clinicians from Uganda and Rwanda completed the intervention and 84 had complete baseline while 77 had complete endline data. Analyses revealed significant improvements in nearly every item on the CRAI-12 scale after the research training program, including increased Self-Efficacy in Designing and Collecting Data (Factor 1); Reporting, Interpreting and Presenting (Factor 2); Conceptualizing and Collaborating (Factor 3); Setting Expectations for Research Staff (part of Factor 4); Describing the Funding Process (Part of Factor 5); and Protecting Study Participants (Factor 6) (all p = < 0.05). The only items that did not improve significantly were Confidence in Asking Staff to Leave the Project Team when Necessary (part of Factor 4) and Locating the Appropriate Grant Application Forms (part of Factor 5).We proposed a conceptual framework outlining the hypothesized pathways through which training and skill development influence research-related career planning and progression.

**Conclusions:**

The research training intervention effectively improved participants’ research competence. These findings underscore the importance of structured research training programs in enhancing research skills. Future research should focus on longitudinal assessments to explore sustained changes and the enduring impact of self-efficacy, outcome expectations (anticipations about career-related consequences), and research-related career goals on career planning and skill development.

**Supplementary Information:**

The online version contains supplementary material available at 10.1186/s12978-025-02047-5.

## Abstract (plain language summary)

In this study, we explored whether a unique training program in Sub-Saharan Africa could help faculty members and clinicians become better at scientific research. This training aimed to help faculty members and clinicians improve confidence and self-efficacy in performing meaningful research. To understand how much the training helped, we asked participants about their research skills before and after they completed the program. We used the Clinical Research Appraisal Inventory (CRAI) -12 scale to measure these changes. We compared their answers before and after the training.

Our results showed that after the training, participants reported improvement in their research competence. These findings suggest that structured training programs may benefit faculty members and clinicians in Sub-Saharan Africa looking to improve their research skills. Individuals may be better equipped to contribute meaningfully to research by focusing on these areas. Further research could explore the long-term effects of such training programs and how they might work in other environments or with different groups of people.

**Supplementary Information:**

The online version contains supplementary material available at 10.1186/s12978-025-02047-5.

## Background

Research training in Sub-Saharan Africa is crucial for strengthening the capacity of healthcare professionals and researchers to address sexual and reproductive health (SRH) challenges within their communities [1]. By improving the quality of research and fostering a culture of innovation, these trainings have the capacity to empower individuals to develop effective, evidence-based interventions tailored to local needs [2]. Additionally, health research publications from Sub-Saharan Africa are under-represented in the academic literature [3, 4] and writing and publishing interventions are an underutilized opportunity for research capacity strengthening [3]. Enhancing research capacity within existing structures offers a practical and economical strategy for cultivating a cadre capable of addressing national and sub-national health needs [1]. While such programs are critical, reproductive health research strengthening programs have often been lacking in rigorous outcome data [5–8].

The Center for International Reproductive Health Training at the University of Michigan (CIRHT-UM) partners with educational institutions in low-resource countries to strengthen human resources for effective contraceptive care (CC), comprehensive abortion care (CAC) and SRH [9]. One arm of the program aims to strengthen and sustain a culture of research in CC/CAC/SRH and bolster faculty research capacity in partner health professional schools by supporting optimal research skills through a systematic, longitudinal approach.

In particular, CIRHT-UM focuses on strengthening within the existing research foundation of academic structures. The research capacity intervention evaluated in this paper leverages a tailored research training program coupled with the disbursement of seed grants to directly fund the initiation of context-specific SRH research endeavors. Seed grants have the potential to nurture preliminary research activities, offering intrinsic motivation and necessary financial support for innovators to explore and address pressing SRH issues [10]. By investing in the local research ecosystem through education and financial assistance, CIRHT-UM intends to bridge the gap between training program completion and implementation of research findings into practice. The ultimate vision is to accomplish sustained improvements in both SRH outcomes and policy development. The goal of this study was to evaluate research competence after participation in the research program.

Our study evaluation was guided by Social Cognitive Career Theory (SCCT), which offers a conceptual framework to explain the development of a person’s career. The theory can facilitate understanding of what makes tailored training opportunities, such as clinical research training, more or less effective in particular individuals [11–13]. Three underlying concepts shaping career behaviors and decisions are self-efficacy, outcome expectations, and personal goals. First, self-efficacy represents individuals’ belief in their ability to achieve career-related tasks and outcomes, significantly impacting goal setting and perseverance [14]. Second, outcome expectations reflect anticipations about career-related consequences, motivating choices such as job satisfaction and advancement [15, 16]. Finally, personal goals rooted in interests and values guide career development and influence decisions. Together, these concepts within SCCT intricately mold individuals’ career paths and overall success.

Guided by the SCCT, our study evaluates self-efficacy in research before and after a two-year research training program for nurses, doctors, and midwives in Uganda and Rwanda by CIRHT-UM. The results of our study will shed light on the program’s effectiveness in enhancing participants’ self-efficacy, shaping positive outcome expectations, and supporting the actualization of career goals within SRH research.

## Methods

### Design

We employed a quasi-experimental, pre-test, and post-test design.

### Setting, sample, and recruitment

The research training program was conducted at the University of Rwanda (campuses in Kigali, Rwamagana, and Huye) along with Gulu and Lira Universities in northern Uganda and their affiliated teaching hospitals. The sample included faculty and clinicians who were enrolled in CIRHT-UM research training programs in Rwanda and Uganda (Gulu and Lira Universities) from departments of nursing, midwifery, and OBGYN. These participants were purposely selected because they all received competitive seed grants as primary investigators for CC/CAC/SRH research projects funded by CIRHT-UM after completing a research webinar series on introductory research topics. Seed grant applications included a detailed research proposal and essay questions related to experience and enthusiasm for scientific research. The applications were peer-reviewed by at least two experts on an international panel and compared using a scoring rubric. All participants in CIRHT-UM research training programs were invited to participate in the assessments via email and WhatsApp.

### Ethics

Institutional Review Board (IRB) exemption approval was obtained prior to beginning the study from the University of Rwanda (CMHS/IRB/210/2022) and the University of Michigan (HUM00203642). IRB approval was obtained from Gulu and Lira Universities (GUREC-2022-252). The IRB in Uganda did not have the provision for exemption. Therefore, full ethical approval was obtained in Uganda. Participants were assured of confidentiality and privacy was respected. No identifiable information was collected.

### Informed consent

An online explanation in English of the purpose of the study was provided prior to the survey, and participants were given an opportunity to ask questions. Written informed consent was obtained in English for all geographic settings.

### Intervention

Prior to the intervention, we conducted a “gap analysis” with participants and institutional leaders wherein we evaluated the training background, resources, and needs as well as the overall research goals of each collaborating institution. The goal of CIRHT-UM gap analysis was to assess researchers’ skills and confidence across various stages of the research cycle, enabling us to customize our support to partners based on their specific needs. Through a survey, we asked researchers to rate their comfort level and proficiency in the following areas: conducting a literature review, mentoring junior faculty and trainees, formulating research questions, selecting an appropriate research design, developing a research protocol, collaborating across disciplines, designing a survey instrument, collecting, managing, and analyzing data, preparing abstracts for meetings, understanding authorship and ethical considerations, choosing suitable journals, publishing articles, and writing grant proposals. This comprehensive survey allowed us to identify areas where researchers excel and where they may require additional support, thus enabling us to provide targeted assistance that maximizes their research potential. Building on the results identified in the gap analysis, our two-year research program consisted of in-person trainings (didactic experiences to instruct participants on research competencies) (*n* = 10), workshops (training activities that emphasized hands-on engagement and collaborative problem-solving) (*n* = 2), webinars (online seminars conducted using video conferencing software) (*n* = 8). A complete list of training topics is displayed in Table 1. All training activities aimed to improve competencies related to engaging with and applying clinical research as part of scientific discovery and evidence-based practice. Topics covered included creating a research question, choosing a research design, searching the literature, primary investigator responsibilities, mentoring [17], grant management, data collection methods, statistical analysis, and implementation science [18].

**Table 1:** Topics covered in the center for international reproductive health training research training programs [6]

Topic	Format of educational activity*	Duration
Before Seed Grant Competition (open to all researchers at the target institutions)		
Developing a Research Question	Remote Webinar	1 h
Conducting a Literature Search	Remote Webinar	1 h
Research Design Types	Remote Webinar	1 h
STATA Statistical Software Training	Remote Webinar	1 h
Advanced STATA Training	Remote Webinar	1 h
Statistical Considerations in Research Proposals	Remote Webinar	1 h
Research Grant Proposal Writing	In-Person Training	8 h
After Seed Grant Award (available to seed grant principal investigators)		
Ethical Conduct of Research	In-Person Training	4 h
Scientific Writing Webinar	Remote Webinar	2 h
Research PI Responsibilities and Grant Management	In-Person Training	8 h
Survey Tool Development	In-Person Training	8 h
Writing and Publishing a Paper for a Peer Reviewed Journal	In-Person Workshop	14 h
Statistics and Software	In-Person Training	8 h
Journal Selection	Remote Webinar	1 h
Qualitative Data Analysis	In-Person Training	8 h
Author Refresher Training	In-Person Workshop	8 h
Qualitative Data Analysis Software Training	In-Person Training	8 h
Quantitative Data Analysis Software Training	In-Person Training	8 h
Post Seed Grant Completion (available to seed grant principal investigators)		
Implementation Science	In-Person Training	8 h
External Grant Writing	In-Person Training	8 h

*Webinars were defined as an online seminar conducted using video conferencing software, which allowed for synchronously live presentation, interactive sessions. Trainings were defined as didactic experiences to instruct participants on research competencies. Workshops were characterized as emphasizing hands-on engagement and collaborative problem-solving

Training on scientific communication topics, such as writing for publication, was provided by CIRHT’s research partner, PREPSS (Pre-Publication Support Service) [19]. The training incorporated SCCT by focusing on building self-efficacy in research appraisal and utilization, enhancing outcome expectations, and promoting the setting of relevant research-related career goals.

#### Measurement instrument and data collection

The Clinical Research Appraisal Inventory-12 (CRAI-12) was used as the primary outcome measure to assess research competence among participants. The CRAI-12 is a validated instrument designed to evaluate various dimensions of research competence, productivity, and professional engagement, referred to as “factors” [20]. The CRAI-12 and CRAI has been used in a limited number of African countries (Cordeiro, Oteng). The scale comprises Likert-type items rated on a ten-point scale, with higher scores indicating greater perceived levels of competence in various aspects of clinical research (Appendix A). Permission to use the CRAI-12 was provided prior to beginning the study by the original developers of the shortened assessment tool.

**Fig. 1:**
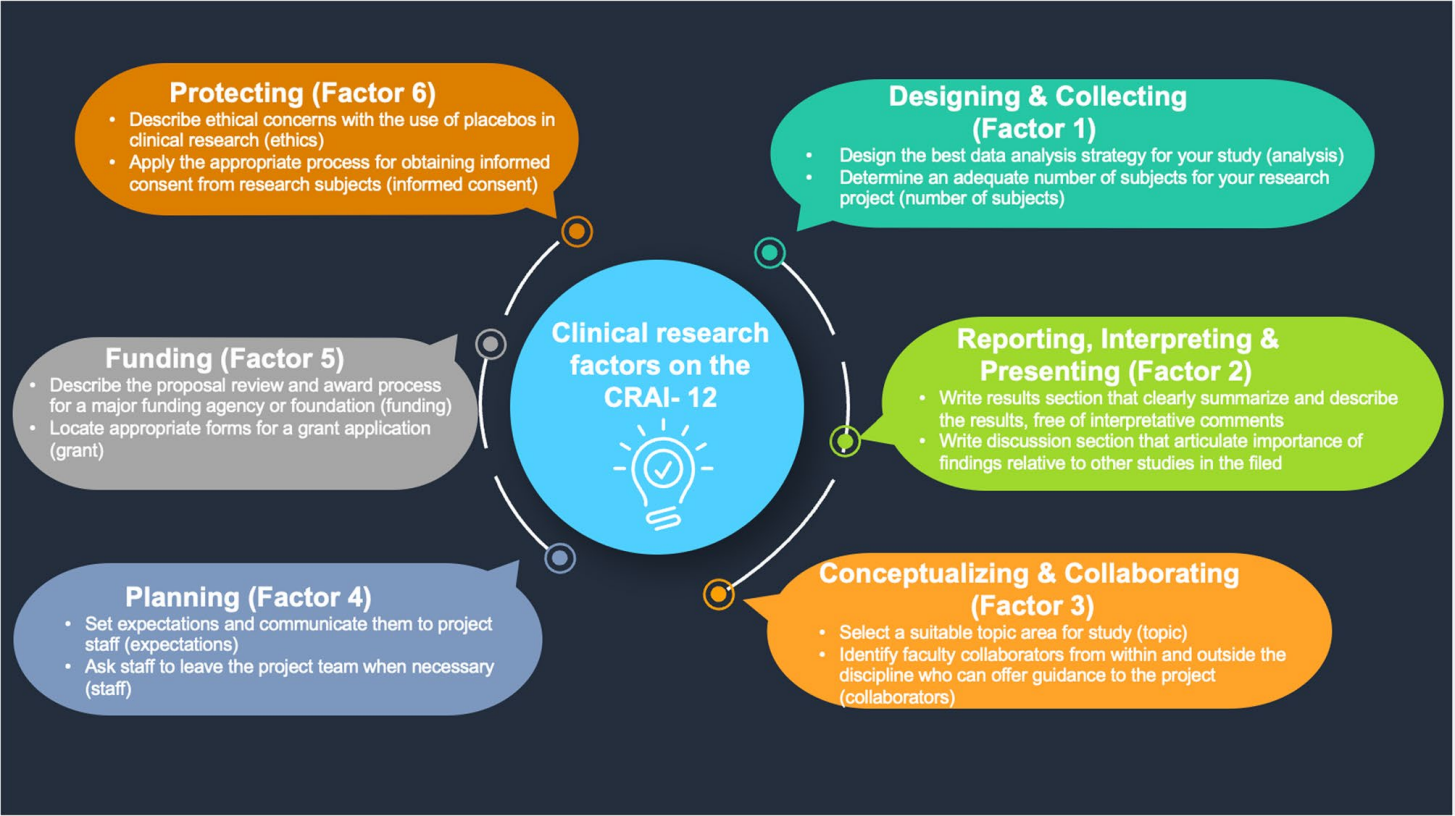
Clinical research factors on the Clinical Research Appraisal Inventory (CRAI)−12 scale [20]

The original extended version of the CRAI was based on the SCCT [16, 20]. As shown in Fig. 1, each factor of the CRAI-12 pertains to a different skill set related to the research process [20]. Factor 1, “Designing and Collecting,” assesses respondents’ proficiency in crafting research protocols and implementing data collection methods to ensure the validity of findings [20]. Factor 2, “Reporting, Interpreting, and Presenting,” assesses individuals’ skills in analyzing data, interpreting results, and effectively communicating research findings to diverse audiences through various mediums [20]. Factor 3, “Conceptualizing and Collaborating,” measures aptitude in generating innovative research ideas and forging collaborations across interdisciplinary teams [20]. Factor 4, “Planning,” evaluates individuals’ capacity to develop comprehensive research plans encompassing study objectives, methodologies, and timelines [20]. Factor 5, “Funding,” scrutinizes individuals’ ability to secure financial support for research endeavors through grant writing and proposal development [20]. Lastly, Factor 6, “Protecting,” evaluates adherence to ethical and regulatory standards, safeguarding human research participants and research integrity [20]. These factors collectively provide a comprehensive framework for evaluating individuals’ competence and contributions in clinical research settings. Together, they encompass a comprehensive view of clinical research capabilities, from conceptualization to ethical conduct. Self-perceived competence in these areas will likely influence a clinician’s engagement with research activities and their application to clinical practice.

The web-based CRAI-12 survey was administered anonymously via Qualtrics through email and WhatsApp. The assessments did not contain questions of a sensitive nature. Demographic information was also linked to the assessments. The baseline CRAI-12 was distributed immediately before the first in-person training session (April 2022 in Rwanda and July 2022 in Uganda). The endline survey was distributed a couple of months after the completion of in-person training (December 2023-January 2024) to gauge retained knowledge and assess the sustainability and long-term impact of the intervention, providing insights for future program improvements. We collected demographic information about sex, age range, country of residence, institutional affiliation, departmental affiliation, and credentials (e.g., lecturer, resident). Figure 1 highlights the dependent variables from our study included in the CRAI-12 survey [20].

### Data analyses

Descriptive statistics were used to summarize demographic characteristics and intervention outcomes in baseline and endline-CIRHT intervention periods. Chi-squared test were used to compare baseline and endline survey results on categorical demographic variables. Kruskal-Wallis tests assessed whether competencies within clinical and scientific research domains changed significantly after the CIRHT intervention. SAS 9.4 was used for statistical analyses, and all tests were two-sided with a 95% confidence level, and significance was considered at *p* = < 0.05.

## Results

### Survey responses

A total of 89 CIRHT seed grant principal investigators completed the intervention (41 from Rwanda and 48 from Uganda). A total of 84 participants completed the baseline survey (all 41 participants from Rwanda and 43 of 48 participants from Uganda) and 77 participants completed the endline survey (all 41 participants from Rwanda and 36 of 48 participants from Uganda) (Table 2).

**Table 2:** Comparison of socio demographic characteristics of respondents who completed the baseline survey and those who completed the endline survey

	Baseline responses (*N* = 84)	Endline responses (*N* = 77)	*P*-value^1^
Country of residence			0.574
Rwanda	41 (48.8%)	41 (53.2%)	
Uganda	43 (51.2%)	36 (46.8%)	
Total	84	77	
Age group			0.508
18–24 years old	2 (2.4%)	1 (1.3%)	
25–34 years old	40 (47.6%)	28 (36.4%)	
35–44 years old	34 (40.5%)	38 (49.4%)	
45–54 years old	6 (7.1%)	9 (11.7%)	
55–64 years old	2 (2.4%)	1 (1.3%)	
Gender			0.938
Female	35 (41.7%)	32 (41.6%)	
Male	48 (57.1)	45 (58.4%)	
Missing	1 (1.2%)	0 (0.0%)	
Highest degree completed			0.782
Diploma	5 (6.0%)	5 (6.5%)	
MD	5 (6.0%)	3 (3.9%)	
Master’s Degree	43 (51.2%)	45 (58.4%)	
PhD	4 (4.8%)	5 (6.5%)	
Undergraduate Degree	27 (32.1%)	19 (24.7%)	
Departmental affiliation			0.555
Obstetrics and Gynecology Department	35 (41.7%)	32 (41.6%)	
School of Nursing and Midwifery	32 (38.1%)	34 (44.2%)	
Other	17 (20.2%)	11 (14.3%)	
Job title			0.257
Lecturer	33 (39.3%)	37 (48.1%)	
Medical Student	1 (1.2%)	5 (6.5%)	
Midwife	8 (9.5%)	8 (10.4%)	
Nurse	5 (6.0%)	2 (2.6%)	
Resident	15 (17.9%)	12 (15.6%)	
Other	22 (26.2%)	13 (16.9%)	

^1^We used a Chi-squared test to compare baseline and endline -test survey respondents on categorical demographic variables. The Mantel-Haenszel chi-squared test was used to compare ordinal variables (age)

### Sociodemographic characteristics

In the endline survey, most of the survey respondents were in the 25–34 (*n* = 28) and 35–44 (*n* = 38) age groups. There were more male (*n* = 45) than female respondents (*n* = 32). The highest degree obtained for most of the respondents was a master’s degree (*n* = 45). Most of the respondents were lecturers (*n* = 37). Table 2 shows the demographic characteristics of respondents. There were no statistically significant differences in demographic characteristics between the group who completed the baseline survey and endline surveys.

### Changes in CRAI-12 scores

At baseline, participants had a modest level of self-efficacy, or confidence, in the six research factors, with mean scores in the 5–8 point (10 points represents the highest confidence) range (Table 3). Following the research training program, scores were in the 7–8 point range (Table 3). In terms of differences between institutions in Rwanda versus Uganda, there were no statistical differences in any factor either at the baseline or endline of the intervention.

**Table 3:** Clinical research appraisal inventory (CRAI)−12 score comparison from baseline to endline of intervention

	Baseline responses (*N* = 84)	Endline responses (*N* = 77)	Mean Difference	Mean Difference Confidence Intervals	*P*-value^1^
**Factor 1: Designing and collecting data**					
**Analysis** Design the best data analysis strategy for your study					
Mean ± SD	5.7 ± 2.17	7.2 ± 1.85	1.4 ± 2.02	**(0.8**,** 2.1)**^2^	**< 0.001**
Median (IQR)	5.5 (2.25)	8.0 (2)			
**Number of subjects** Determine an adequate number of subjects for your research project					
Mean ± SD	6.85 ± 2.16	7.8 ± 1.65	1.0 ± 1.94	**(0.4**,** 1.6)**	**0.004**
Median (IQR)	7.0 (3)	8.0 (2)			
**Factor 2: Reporting, interpreting and presenting**					
**Results** Write the results section of a research paper that clearly summarizes and describes the results, free of interpretative comments					
Mean ± SD	6.5 ± 2.29	7.9 ± 1.67	1.4 ± 2.02	**(0.7**,** 2.0)**	**< 0.001**
Median (IQR)	7.0 (3)	8.0 (2)			
**Discussion** Write a discussion section for a research paper that articulates the importance of your findings relative to other studies in the field					
Mean ± SD	6.6 ± 2.06	7.8 ± 1.67	1.2 ± 1.88	**(0.6**,** 1.7)**	**< 0.001**
Median (IQR)	7.0 (3)	8.0 (2)			
**Factor 3: Conceptualizing and collaborating**					
**Topic**: Select a suitable topic area for study					
Mean ± SD	7.7 ± 1.66	8.5 ± 1.33	0.8 ± 1.51	**(0.3**,** 1.3)**	**0.002**
Median (IQR)	8.0 (3)	9.0 (2)			
**Collaborators** Identify faculty collaborators from within and outside the discipline who can offer guidance to the project					
Mean ± SD	7.4 ± 2.09	8.56 ± 1.45	1.2 ± 1.82	**(0.6**,** 1.8)**	**< 0.001**
Median (IQR)	8.0 (3)	9.0 (2)			
**Factor 4: Planning**					
**Expectations** Set expectations and communicate them to project staff					
Mean ± SD	7.3 ± 2.02	8.3 ± 1.51	1.0 ± 1.79	**(0.4**,** 1.5)**	**0.002**
Median (IQR)	7.5 (3)	8.0 (2)			
**Staff** Ask staff to leave the project team when necessary					
Mean ± SD	6.6 ± 2.40	7.2 ± 2.04	0.6 ± 2.24	(−0.11, 1.29)	0.153
Median (IQR)	7.0 (3)	8.0 (2)			
**Factor 5: Funding**					
**Funding process** Describe the proposal review and award process for a major funding agency or foundation					
Mean ± SD	5.9 ± 2.35	7.1 ± 1.86	1.2 ± 2.13	(**0.6**,** 1.9)**	**< 0.001**
Median (IQR)	6.0 (3)	8.0 (2)			
**Grant application** Locate appropriate forms for a grant application					
Mean ± SD	6.3 ± 2.44	7.1 ± 1.70	0.8 ± 2.12	**(0.2**,** 1.5)**	**0.053**
Median (IQR)	7.0 (3)	7.0 (2)			
**Factor 6: Protecting**					
**Ethics** Describe ethical concerns with the use of placebos in clinical research					
Mean ± SD	6.0 ± 2.70	7.1 ± 2.26	1.1 ± 2.50	**(0.3**,** 1.9)**	**0.011**
Median (IQR)	6.0 (4)	7.0 (3)			
**Informed consent** Apply the appropriate process for obtaining informed consent from research subjects					
Mean ± SD	8.0 ± 2.02	8.9 ± 1.30	0.9 ± 1.72	**(0.4**,** 1.5)**	**0.001**
Median (IQR)	8.0 (2.25)	9.0 (2)			

^1^We used a Kruskal-Wallis test to identify whether the mean change in Factor scores between baseline and endline was significantly different from 0

^2^Bolded values indicate significant mean changes between baseline and endline Factor scores

Significant improvements were identified in mean post-training scores for nearly all Factors (Table 3). Significant increases in both components of Factor 1, Designing and Collecting Data (Analysis and Number of Subjects) and both components of Factor 2 (Reporting, Interpreting, and Presenting the Results and Discussion) suggest a heightened self-efficacy in both the methodological and communicative aspects of research resonating with the premise of SCCT, that self-efficacy is foundational for performing specific tasks effectively.

Significant improvements in the mean questionnaire scores from baseline to endline were observed in both components of Factor 3, (mean difference in Selecting a Topic 0.8 [0.3, 1.3] and Identifying Collaborators 1.2 [0.6, 1.8]) and significant increase in Setting Expectations for Project Staff (one component of Factor 4) (mean difference 1.0 [0.4, 1.5] align with the SCCT in that these changes might influence researchers’ outcome expectations. Participants may thus believe that their research efforts will lead to meaningful contributions to clinical practice.

Finally, we noted significant increases in one component of Factor 5 (mean difference in Describing the Grant Funding Process 1.2 [0.6, 1.9]) and significant increases in both components of Factor 6 (mean difference in Protecting Study Participants via Ethics 1.1 [0.3, 1.9] and Informed Consent 0.9 [0.4, 1.5]).

### Operationalization of the social cognitive career theory (SCCT) for clinical research training program and appraisal

**Fig. 2:**
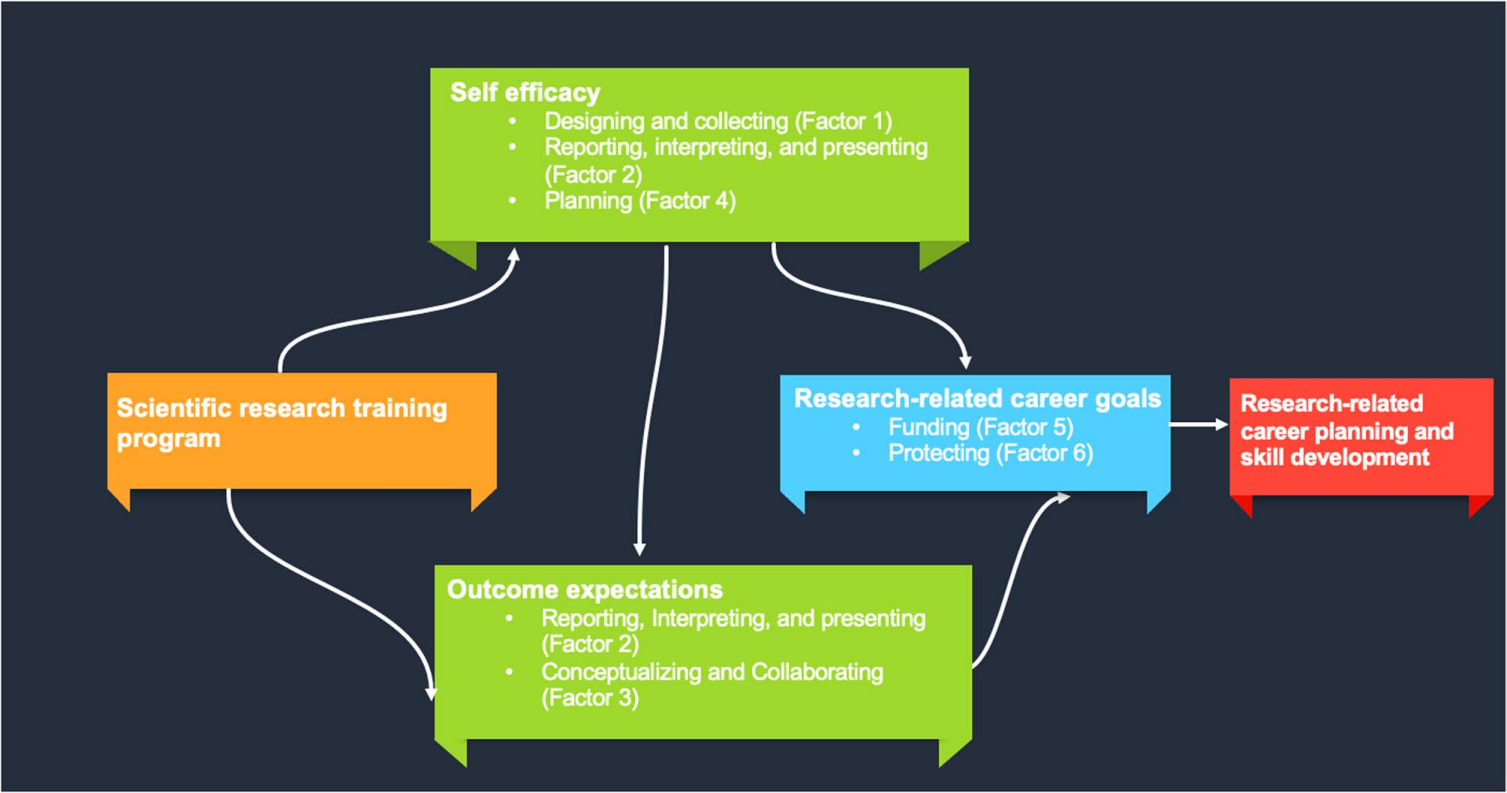
Adapted conceptual framework integrating findings from the Clinical Research Appraisal Inventory-12 survey within the *Social Cognitive Career Theory* [11, 12, 20]

By understanding topics covered in the CRAI-12, faculty and clinician researchers can develop their research appraisal skills, aligning with SCCT’s emphasis on self-efficacy, outcome expectations, and goal setting to foster career development and advancement in research. Figure 2 displays an adapted conceptual framework showing how we hypothesize the research training program impacts factors of the CRAI-12 scale related to SCCT constructs of self-efficacy (Factor 1, designing and collecting; Factor 2, reporting, interpreting, and presenting; Factor 4, planning), outcome expectations (Factor 2, reporting, interpreting, and presenting; Factor 3, conceptualizing and collaborating), and research-related career goals (Factor 5, funding; Factor 6, protecting) ultimately influencing research-related career planning and skill development. Our adapted conceptual framework outlines the hypothesized pathways through which training and skill development influence research-related career planning and skill development. A comprehensive understanding of self-efficacy, outcome expectations, and goals related to the CRAI-12 creates a foundation for clinicians to build a research-oriented career path, furthering their contributions to evidence-based practice and healthcare improvements.

## Discussion

Study findings suggest that the targeted international reproductive health training program by CIRHT helps faculty members and clinicians gain confidence and self-efficacy in their ability to perform meaningful research in SRH. The training positively influenced participants’ confidence in using clinical research in practice, as indicated by significant post-intervention improvements in most of the CRAI-12 scores. Endline scores identified topics on which the learners needed additional time-limited support to achieve program goals. Using scales like the CRAI-12 in reproductive health research allows for a comprehensive assessment of research competence and training effectiveness, ultimately contributing to improving research practices and outcomes in this critical healthcare field. Results can potentially be used to advocate for research curriculum policy changes within or across departments.

Several facets of the CIRHT-UM research training program may have contributed to the significant gains in research competencies. The participants received seed grants to apply new research skills in conjunction with the trainings and webinars described. In particular, these projects required multidisciplinary teams, which encouraged skill-building in communication and collaboration, as is queried in Factor 3 (conceptualizing and collaborating). The research trainings very intentionally cover the lifecycle of a research project to ensure support and skills training at each phase of a research project. In addition, we emphasized mentorship from the start, and each participant selected a senior faculty researcher as a mentor. Mentors received separate mentorship skills training to strengthen local support and continually reinforce the research education provided by CIRHT-UM. Furthermore, the significant increases in one component of Factor 5 (Describing the Grant Funding Process) and both components of Factor 6 (Protecting Study Participants via Ethics and Informed Consent) indicate that participants felt more adept at navigating funding processes and ethical considerations and anticipated that these capabilities could lead to more successful research engagement and better patient outcomes.

The observed enhancements in CRAI-12 scores indicate an increased likelihood of clinicians setting and committing to research-related career goals, such as pursuing grants or engaging in collaborative studies, aligning with the SCCT’s assertion that self-efficacy and positive outcome expectations are instrumental in goal-setting processes.

Enhanced self-efficacy, an integral component of SCCT, may underlie the improvement in CRAI-12 scores after the implementation of seed grants and completion of the research training program, with clinicians demonstrating increased confidence in interpreting and applying research findings. Consistent with SCCT, heightened outcome expectations—beliefs that training leads to better presentation of results and discussion of research findings—also appeared to contribute to the observed changes. The training’s influence on research-related career goal setting was also evident, with participants reporting clearer objectives regarding grant funding and ethical research engagement. Research-related career goal setting based on these motivational constructs enables faculty and clinicians to outline and adhere to clear steps in their professional development, aligning their day-to-day activities with long-term career aspirations in research. Overall, the results affirm SCCT’s utility in understanding the professional development of faculty members and clinicians in Sub-Saharan Africa.

This aligns well with existing literature on improving SRHR research capabilities. The training’s success in enhancing participants’ self-efficacy supports SCCT, which underscores self-efficacy and positive outcome expectations in goal setting and career development. Our findings are consistent with a systematic review by Perrotta et al. [21], which determined that remote training strengthens capacity in SRH research. Well-organized courses with reasonable workloads were key factors in enabling the acquisition of new research skills [21]. The structured use of webinars also contributed to building faculty and clinician confidence and self-efficacy in research undertakings [21].

The practical application of skills through seed grants further enhances the training’s impact by allowing participants to immediately apply their newfound abilities within a multidisciplinary team framework. This hands-on approach reinforces learning and skill acquisition, ensuring that the training is not only theoretical but also practical. Ahmed et al. affirm the legitimacy of such strategies, emphasizing the benefits of cross-disciplinary teamwork in research settings [22]. This also aligns with assertions by Akinyemi et al. that cross-disciplinary teamwork elevates the research experience and fosters collaboration, innovation, and a more comprehensive understanding of diverse perspectives within SRHR research, providing a richer and more inclusive research environment [23].

Mentorship also plays a pivotal role in our training program and is strongly supported by existing literature. The program’s emphasis on mentorship is designed to facilitate skill acquisition and professional development, with local mentors providing crucial guidance and support, echoes the Fogarty International Center studies in Kenya and Uganda reported by Bennet et al. [24], which highlighted the vital role of local mentors in skill transmission and the cultivation of a generative cycle of mentorship. This comprehensive approach ensures that the benefits of research training extend beyond individual competencies to influence broader systemic improvements in health practices while promoting a sustainable culture of knowledge sharing and continuous learning in the medical community [24].

Furthermore, the program’s outcomes can be strategically utilized to advocate for research curriculum policy changes. By highlighting the effectiveness of our interventions, we contribute to systemic improvements within academic departments, promoting an organizational culture that supports SRH research empowerment. Addressing systemic disparities is crucial for creating equitable research environments, as described by Witteman et al. [25]. Our initiative aligns with calls for reforms that ensure fair opportunities, resources, and recognition for all researchers, promoting a culture of inclusivity and equity.

Finally, supporting underrepresented groups, such as women in global health authorship, is a core aspect of our program. Mentoring in publishing provides much-needed support of women, who are under-represented in global health authorship [26]. Additionally, empowering these groups addresses ongoing challenges documented in the literature and enriches the academic and research landscape by bringing diverse voices and perspectives to the forefront [27]. This inclusivity aligns with broader global goals of equity in healthcare, ensuring that research outputs are representative of the populations being studied and that all researchers have the opportunity to contribute and thrive in the field of SRHR [28].

### Strengths and limitations

The CRAI-12 survey addresses several vital aspects of the clinical research process, comprehensively evaluating clinicians’ self-perceived research skills and attitudes toward research. In terms of limitations, as with any survey based on self-reporting, there is potential for response bias where respondents might either overestimate or underestimate their capabilities, impacting the reliability of the data. Furthermore, certain factors on the CRAI-12 might vary depending on the specific research environment and resources available across settings, such as varying levels of institutional support, access to mentorship, infrastructure, and the nature of research projects, all of which can influence perceptions of competence and opportunities in clinical research.

## Conclusions

The research training intervention effectively improved participants’ research competence and interest in conducting research, altering the perception of research training within clinical research. The significantly improved research competencies across the CRAI-12 factors demonstrate a potential comprehensive growth in skills that could extend beyond self-efficacy to encapsulate enhanced outcome expectations and perhaps even a greater propensity to establish and pursue research-oriented career goals. These findings highlight the significance of structured research training programs in advancing research-related career planning and skill development. As a result of the enhanced research competence, the visibility of the research and the output of the participating institutions are expected to increase, contributing to the body of knowledge in SRH and enriching the literature. Future studies are being planned to concentrate on longitudinal assessments to investigate sustained changes and the lasting effects of self-efficacy, outcome expectations, and research-related career objectives on career planning and skill advancement. This timeframe will allow us to assess any lasting behavioral or systemic changes and understand any late-emerging outcomes that might not be evident immediately after the intervention.

## Supplementary Information

Supplementary Material 1.

## Data Availability

No datasets were generated or analysed during the current study.

## Abbreviations

CAC: Comprehensive abortion care
CC: Contraceptive care
CIRHT-UM: Center for International Reproductive Health Training at the University of Michigan
CRAI: Clinical Research Appraisal Inventory
IRB: Institutional Review Board
PREPSS: Pre-Publication Support Service
SCCT: Social Cognitive Career Theory
SRH: Sexual and reproductive health
