# Supplementary material for: Impact of a comprehensive two-year research capacity intervention with sexual and reproductive health researchers in Sub-Saharan Africa
Source: Reprod Health. 2025 May 31;22:94. doi: 10.1186/s12978-025-02047-5 (PMC12126909; doi:10.1186/s12978-025-02047-5)
Supplement: Supplementary file 1 — Supplementary Material 1. [file 12978_2025_2047_MOESM1_ESM.docx]

**Appendix A: 12- Item Clinical Research Appraisal Inventory (CRAI-12)**

**INSTRUCTIONS:** The following items are tasks related to performing clinical research. Please indicate your ability to successfully perform each task by selecting a single number from zero to ten that best describes your level of confidence. The phrases next to the numbers (0=No Confidence and 10=Total Confidence) are only guides. You can use these numbers or any of the numbers in between to describe your level of confidence. We would like to know how confident you are that you can successfully perform these tasks today.

**Designing and collecting ________________________________________________________**

1. Design the best data analysis strategy for your study.

☐ No Confidence ☐ 1 ☐ 2 ☐ 3 ☐ 4 ☐ 5 ☐ 6 ☐ 7 ☐ 8 ☐ 9 ☐ Total Confidence

1. Determine an adequate number of subjects for your research project.

☐ No Confidence ☐ 1 ☐ 2 ☐ 3 ☐ 4 ☐ 5 ☐ 6 ☐ 7 ☐ 8 ☐ 9 ☐ Total Confidence

**Reporting, interpreting and presenting ____________________________________________**

1. Write the results section of a research paper that clearly summarizes and describes the results, free of interpretative comments.

☐ No Confidence ☐ 1 ☐ 2 ☐ 3 ☐ 4 ☐ 5 ☐ 6 ☐ 7 ☐ 8 ☐ 9 ☐ Total Confidence

1. Write a discussion section for a research paper that articulates the importance of your findings relative to other studies in the field.

☐ No Confidence ☐ 1 ☐ 2 ☐ 3 ☐ 4 ☐ 5 ☐ 6 ☐ 7 ☐ 8 ☐ 9 ☐ Total Confidence

**Conceptualizing and collaborating ________________________________________________**

1. Select a suitable topic area for study.

☐ No Confidence ☐ 1 ☐ 2 ☐ 3 ☐ 4 ☐ 5 ☐ 6 ☐ 7 ☐ 8 ☐ 9 ☐ Total Confidence

1. Identify faculty collaborators from within and outside the discipline who can offer guidance to the project.

☐ No Confidence ☐ 1 ☐ 2 ☐ 3 ☐ 4 ☐ 5 ☐ 6 ☐ 7 ☐ 8 ☐ 9 ☐ Total Confidence

**Planning _____________________________________________________________________**

1. Set expectations and communicate them to project staff.

☐ No Confidence ☐ 1 ☐ 2 ☐ 3 ☐ 4 ☐ 5 ☐ 6 ☐ 7 ☐ 8 ☐ 9 ☐ Total Confidence

1. Ask staff to leave the project team when necessary.

☐ No Confidence ☐ 1 ☐ 2 ☐ 3 ☐ 4 ☐ 5 ☐ 6 ☐ 7 ☐ 8 ☐ 9 ☐ Total Confidence

**Funding ______________________________________________________________________**

1. Describe the proposal review and award process for a major funding agency or foundation.

☐ No Confidence ☐ 1 ☐ 2 ☐ 3 ☐ 4 ☐ 5 ☐ 6 ☐ 7 ☐ 8 ☐ 9 ☐ Total Confidence

1. Locate appropriate forms for a grant application.

☐ No Confidence ☐ 1 ☐ 2 ☐ 3 ☐ 4 ☐ 5 ☐ 6 ☐ 7 ☐ 8 ☐ 9 ☐ Total Confidence

**Protecting ____________________________________________________________________**

1. Describe ethical concerns with the use of placebos in clinical research.

☐ No Confidence ☐ 1 ☐ 2 ☐ 3 ☐ 4 ☐ 5 ☐ 6 ☐ 7 ☐ 8 ☐ 9 ☐ Total Confidence

1. Apply the appropriate process for obtaining informed consent from research subjects.

☐ No Confidence ☐ 1 ☐ 2 ☐ 3 ☐ 4 ☐ 5 ☐ 6 ☐ 7 ☐ 8 ☐ 9 ☐ Total Confidence
